# A novel Modulator of Ring Stage Translation (MRST) gene alters artemisinin sensitivity in *Plasmodium falciparum*

**DOI:** 10.1128/msphere.00152-23

**Published:** 2023-05-23

**Authors:** Caroline Simmons, Justin Gibbons, Chengqi Wang, Camilla Valente Pires, Min Zhang, Faiza Siddiqui, Jenna Oberstaller, Debora Casandra, Andreas Seyfang, Liwang Cui, Thomas D. Otto, John H. Adams

**Affiliations:** 1 Center for Global Health and Infectious Diseases Research and USF Genomics Program, College of Public Health, University of South Florida, Tampa, Florida, USA; 2 Department of Molecular Medicine, Morsani College of Medicine, University of South Florida, Tampa, Florida, USA; 3 Department of Internal Medicine, Morsani College of Medicine, University of South Florida, Tampa, Florida, USA; 4 Institute of Infection, Immunity, and Inflammation, College of Medical, Veterinary and Life Sciences, University of Glasgow, Glasgow, UK; Weill Cornell Medicine, New York, New York, USA

**Keywords:** malaria, antimalarial agents, drug resistance mechanisms, gene annotation

## Abstract

The implementation of artemisinin (ART) combination therapies (ACTs) has greatly decreased deaths caused by *Plasmodium falciparum* malaria, but increasing ACT resistance in Southeast Asia and Africa could reverse this progress. Parasite population genetic studies have identified numerous genes, single-nucleotide polymorphisms (SNPs), and transcriptional signatures associated with altered artemisinin activity with SNPs in the Kelch13 (K13) gene being the most well-characterized artemisinin resistance marker. However, there is an increasing evidence that resistance to artemisinin in *P. falciparum* is not related only to K13 SNPs, prompting the need to characterize other novel genes that can alter ART responses in *P. falciparum*. In our previous analyses of *P. falciparum piggyBac* mutants, several genes of unknown function exhibited increased sensitivity to artemisinin that was similar to a mutant of K13. Further analysis of these genes and their gene co-expression networks indicated that the ART sensitivity cluster was functionally linked to DNA replication and repair, stress responses, and maintenance of homeostatic nuclear activity. In this study, we have characterized PF3D7_1136600, another member of the ART sensitivity cluster. Previously annotated as a conserved *Plasmodium* gene of unknown function, we now provide putative annotation of this gene as a Modulator of Ring Stage Translation (MRST). Our findings reveal that the mutagenesis of MRST affects gene expression of multiple translation-associated pathways during the early ring stage of asexual development via putative ribosome assembly and maturation activity, suggesting an essential role of MRST in protein biosynthesis and another novel mechanism of altering the parasite’s ART drug response.

**IMPORTANCE:**

*Plasmodium falciparum* malaria killed more than 600,000 people in 2021, though ACTs have been critical in reducing malaria mortality as a first-line treatment for infection. However, ACT resistance in Southeast Asia and emerging resistance in Africa are detrimental to this progress. Mutations to Kelch13 (K13) have been identified to confer increased artemisinin tolerance in field isolates, however, genes other than K13 are implicated in altering how the parasite responds to artemisinin prompts additional analysis. Therefore, in this study we have characterized a *P. falciparum* mutant clone with altered sensitivity to artemisinin and identified a novel gene (PF3D7_1136600) that is associated with alterations to parasite translational metabolism during critical timepoints for artemisinin drug response. Many genes of the *P. falciparum* genome remain unannotated, posing a challenge for drug–gene characterizations in the parasite. Therefore, through this study, we have putatively annotated PF3D7_1136600 as a novel MRST gene and have identified a potential link between MRST and parasite stress response mechanisms.

## INTRODUCTION

Malaria afflicted about 247 million people and killed about 619,000 people in 2021, with increasing *Plasmodium falciparum* artemisinin resistance and artemisinin combination therapy (ACT) failure in Southeast (SE) Asia threatening to exacerbate its clinical burden (1). Alarmingly, studies have revealed the emergence of ACT resistance in Africa prompting the need to annotate novel genetic factors associated with altered artemisinin sensitivity to better track resistance in the field (2). Understanding ART resistance is complicated by the multi-pronged artemisinin mechanism of action, which includes damage to DNA, disruption of proteasome activity, accumulation of damaged/misfolded proteins leading to disruption of proteostasis, disruption to mitochondrial activity, free-radical-mediated oxidative stress, and disruption of hemoglobin digestion (3 - 9).

Mutations in the Kelch13 (K13) gene are the most well-characterized marker for artemisinin resistance, with the highest prevalence in SE Asia and recent emergence in Africa. Mutations to K13 have been linked to reduced binding to and polyubiquitination of phosphatidylinositol 3-kinase, increasing kinase levels and PI3P product, a key mediator of artemisinin resistance (6). Additionally, mutations to K13 are associated with disrupted nuclear metabolism, augmented antioxidant and damage-sensing properties of the mitochondria, and inactivation of K13 has led to cell cycle arrest at the ring stage promoting cell quiescence under ART exposure (3, 7, 8, 10 - 13). Recent studies have also identified links between K13 and altered endocytosis of host red blood cell cytosol and hemoglobin that can alter the rate of hemoglobin digestion and artemisinin activation, mediated by the K13 endocytosis complex (14). Several key mutations to the K13 propeller domain have been identified in the field to cause partial artemisinin resistance, including C580Y, Y493H, R539T, and I543T (6, 15).

While distinct single-nucleotide polymorphisms (SNPs) in K13 have previously been identified and studied, ongoing research posits a more interactive network between genetic makeup, transcriptional responses, and metabolic activities to play a combined role in the introduction, spread, and maintenance of artemisinin treatment failure in Asia and Africa (16 - 20). *P. falciparum* has demonstrated an ability to elongate its ring stage of development during the intraerythrocytic development cycle (IDC), thus leading to a longer period of no hemoglobin uptake and digestion, thereby decreasing artemisinin activation (7, 10, 11, 16). Recent studies from our laboratory and other research groups have also shown that *P. falciparum* appears to utilize essential and conserved pathways associated with its innate fever response mechanism to withstand artemisinin exposure, including heat shock, proteasome activity, protein folding, and apicoplast-derived isoprenoid biosynthesis (5, 9, 21 - 23). Proper balance of proteostasis under artemisinin-mediated oxidative stress is necessary to respond to a buildup of damaged, misfolded, and unfolded proteins, with artemisinin-tolerant parasites upregulating chaperones to stabilize proteins and upregulating the core proteasome and vesicular trafficking to degrade or eliminate non-reparable proteins (5, 9, 21 - 23).

Previous genome-wide association studies (GWAS) and transcriptome-wide association studies (TWAS) have shown that K13 mutation-mediated resistance is more likely to occur with upregulated unfolded protein response progression (to maintain a proper balance of proteostasis under artemisinin exposure) and delayed IDC progression (elongated ring stage) (18, 24). GWAS and TWAS studies have additionally noted high transcriptional diversity of *P. falciparum* isolates in areas with high artemisinin resistance and that parasite physiology, and thereby phenotype, is controlled by specific transcriptional profiles and genetic patterns, including genes regulating metabolic growth (glycolysis), response to proteotoxic stress, redox metabolism, vesicular trafficking, and host cytoplasm remodeling (16, 25). Lastly, GWAS/TWAS studies have identified novel genetic and transcriptional variations of genes not related to K13 that confer artemisinin tolerance *in vivo* and *in vitro*, including ubiquitin carboxyl-terminal hydrolase 1 (UBP-1), autophagy-related protein 18 (ATG18), and coronin (26 - 30). Though ongoing studies have hypothesized a link between the interactions of these genes with K13 to illicit a complex artemisinin-resistant response, the ability of *P. falciparum* to develop artemisinin tolerance independent of K13 mutations stresses additional transcriptomic studies.

With increasing resistance to artemisinin and the variety of mechanisms not linked to K13 that *P. falciparum* can undergo to become artemisinin tolerant, characterization of novel artemisinin-linked genes and the transcriptomic patterns associated with these genes are needed to address the need for novel antimalarials targeting key vulnerable parasite-specific processes. Chemogenomic profiling of single-insertion *piggyBac* transposon mutants has been integral in defining drug–gene interactions based on *piggyBac* mutant drug response profiles (31, 32). We previously validated the usefulness of this process in identifying novel artemisinin-linked genes through annotation of the artemisinin sensitivity cluster (ASC), a group of seven genes with associated *piggyBac* mutants exhibiting an increased sensitivity to artemisinin, including a *piggyBac* mutant of K13 (31). Transcriptomic analysis of this K13 *piggyBac* mutant showed significant dysregulation of K13 gene expression, correlating to dysregulation of DNA replication and repair processes, consistent with Kelch13’s role as a stress response regulator (12). In this study, we further define the transcriptome of an ASC *piggyBac* mutant of a Conserved *Plasmodium* Gene of Unknown Function, which we have now annotated as a Modulator of Ring Stage Translation (MRST, PF3D7_1136600). We show significant dysregulation of a variety of translation-associated pathways in the MRST mutant due to MRST downregulation, linking this gene to proper translational gene expression in *P. falciparum*.

## RESULTS

### Artemisinin-sensitive phenotype of the MRST *piggyBac* mutant

**Fig 1:**
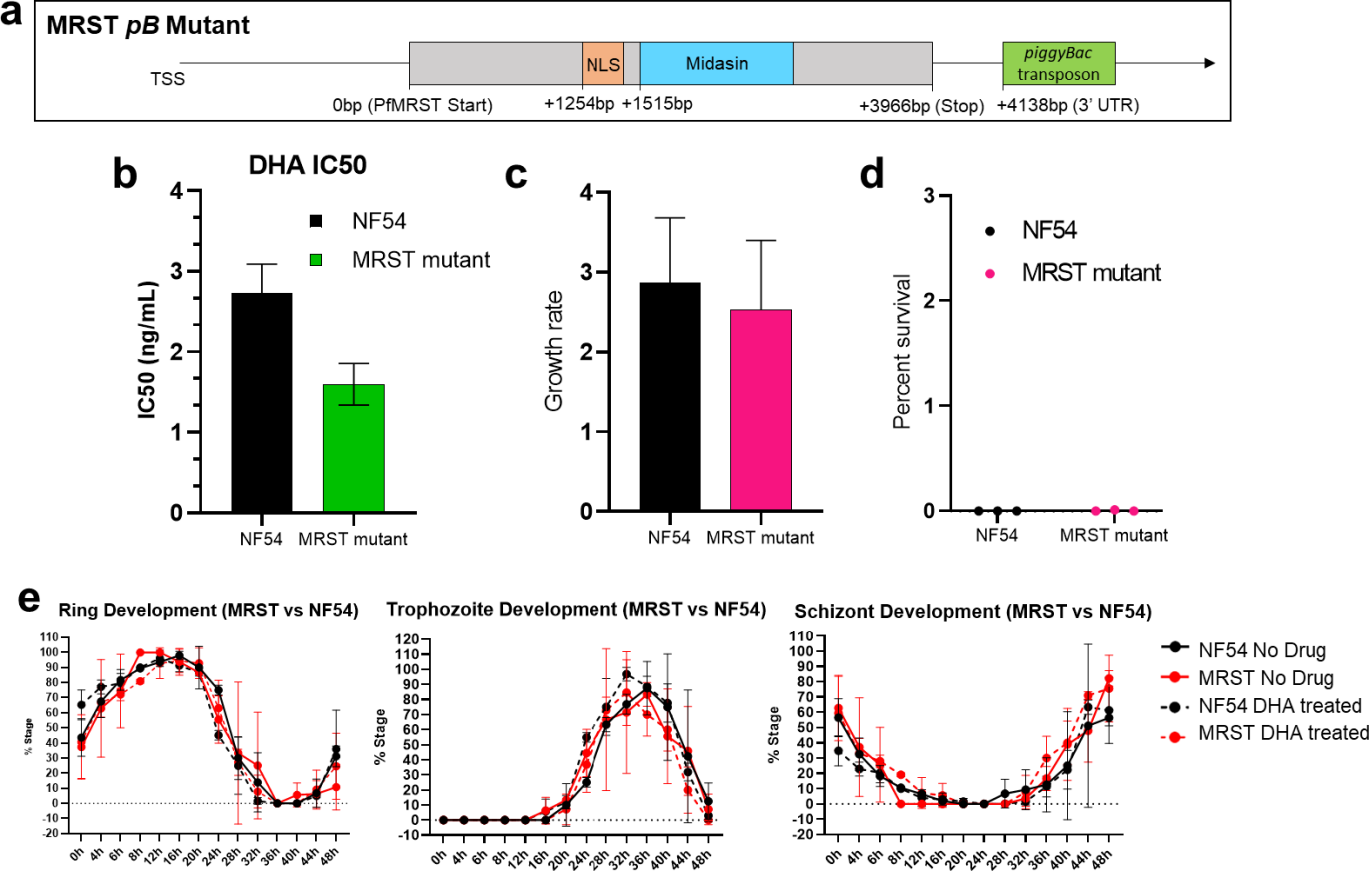
Phenotype of the MRST mutant. (**a**) The MRST mutant contains a *piggyBac* insertion in the annotated 3′ UTR of PF3D7_1136600, which we have termed a Modulator of Ring Stage Translation (MRST) (33, 34). This insertion lies +4138 bp downstream of the start codon. Because MRST lacks functional annotation, bioinformatic analysis identified a putative nuclear localization signal +1,254 bp downstream of the start codon, along with a predicted midasin protein linker and disordered region homology, associated with ribosome nuclear export and maturation, +1,515 bp downstream of start codon. NLS and midasin domain were predicted in Prosite ExPasy and NCBI Blast, respectively. (**b**) SYBR Green I malaria drug sensitivity assay was performed to validate the artemisinin-sensitive phenotype of the mutant. IC50 values averaged across six bio-replicates of the NF54 parent and MRST mutant plotted via bar graph and analyzed via Mann–Whitney test (*P*-value: 0.002). Data were analyzed and plotted via GraphPad Prism. Ring stage survival assay was also performed to validate MRST mutant DHA sensitivity, demonstrating that NF54 and MRST mutant exhibited similar growth rates under normal conditions (**c**). Both the mutant and NF54 had decreased survival under a DHA drug pulse (0.004% survival for mutant and 0% survival for NF54) (**d**). Progression of ring, trophozoite, and schizont cell cycle progression was analyzed with and without a 6-hour DHA drug pulse (**e**). Line graphs show average percentages of each stage every 4 hours during the 48-hour life cycle in drug- and non-drug-treated NF54 and MRST mutant clones. We observed no significant changes in asexual cell cycle development between NF54 and mutant with and without DHA drug exposure, pointing to metabolic changes playing a role in sensitive phenotype (significant *P*-value < 0.05). Stage progression was analyzed via microscopy, with data analysis and statistics (Fisher’s exact test of cell counts) performed in GraphPad Prism and R, respectively. NLS, nuclear localization signal; IC50, half maximal inhibitory concentration; DHA, dihydroartemisinin.

The mutant selected for this study contains a 3′ UTR *piggyBac* transposon insertion +4,138 bp downstream of the annotated start codon of a currently annotated conserved *Plasmodium* gene of unknown function, PF3D7_1136600 (Fig. 1a) (33, 34). WGS was performed using the Illumina NextSeq 550 system for the NF54 parent clone and the PF3D7_1136600 *piggyBac* mutant to both confirm the *piggyBac* insertion site location in the mutant and to ensure that additional mutations were not present in the NF54 and PF3D7_1136600 mutants used in this study (WGS data available via the NCBI Sequence Read Archive [SRA] with the BioProject ID PRJNA947639). We confirmed no additional mutations in our NF54 and mutant and identified the correct *piggyBac* transposon insertion in the annotated 3′ UTR of PF3D7_1136600 in the mutant, +4,138 bp downstream of the start codon (33, 34).

PF3D7_1136600 remains a conserved *Plasmodium* gene with a lack of functional annotation, thus prompting our decision to perform a preliminary bioinformatic analysis of the PF3D7_1136600 protein sequence to determine putative conserved domains and protein characteristics. We first identified via NCBI Blast partial similarity to the linker and disordered region of the *Saccharomyces cerevisiae* midasin protein (MDN1p, YLR106p), a 4,910 amino acid-long AAA+ ATPase family nuclear chaperone protein required for the maturation and nuclear export of ribosome subunits (Fig. 1a) (35, 36). Similarity spanned the 505-951aa positions in PF3D7_1136600 and the 3874-4305aa position in the midasin gene (*E*-value: 3.97e-03). This ~440-residue similarity in the 4,910-residue midasin protein represents only a portion of the midasin linker domain (which provides a structural role in the midasin protein while also predicted to act as a hinge or protein-binding site) and disordered domain, thus partially linking PF3D7_1136600 to putative ribosome maturation processes and requiring further investigation (36). A nuclear localization signal was also bioinformatically predicted via ExPasy Prosite located 418aa–432aa downstream of the start codon, bioinformatically supporting a nuclear-associated function for PF3D7_1136600 (Fig. 1a) (37). We herein refer to PF3D7_1136600 as an MRST based on our preliminary bioinformatic partial characterization and our downstream analysis of the MRST mutant asexual blood stage transcriptome.

We next sought to further characterize the ART response phenotype of the MRST mutant through additional drug sensitivity assays. SYBR Green I malaria drug sensitivity assay was used to validate the dihydroartemisinin IC50 of the MRST mutant clone as previously published (mutant IC50 value: 1.6 ng/mL; NF54 IC50 value: 2.7 ng/mL, *P*-value: 0.002165, averaged across six bio-replicates) (Fig. 1b; Data Set S1 Tab 1) (31, 38, 39). Further, RSA identified an average percent survival rate of 0.004% for the MRST mutant under a DHA drug pulse and an average percent survival rate of 0% for the NF54 parent clone, indicating DHA drug sensitivity of the mutant and NF54 (Fig. 1c and d; Data Set S1 Tab 2) (40). Tolerance to artemisinin particularly occurs during the ring stage of the IDC, with artemisinin-resistant (ART-R) parasite clones being shown to elongate its ring stage progression (10, 11). As ring stage is the least metabolically active stage and thereby the least sensitive to DHA exposure compared to trophozoite, schizont, and merozoite stages, we sought to determine whether ring stage progression is altered in the artemisinin-sensitive (ART-S) MRST mutant by characterizing IDC development under a 6-hour DHA drug pulse, similar to the RSA (10, 11, 19). We observed no significant changes in MRST development of ring, trophozoite, and schizont stages with and without DHA drug pulse compared to the NF54 parent clone, indicating that the ART-S phenotype may not be directly attributed to the intraerythrocytic ring stage development (*P*-value > 0.05) (Fig. 1e; Fig. S1; Data Set S1 Tab 3).

### Early ring stage gene expression is dysregulated due to the MRST gene disruption

**Fig 2:**
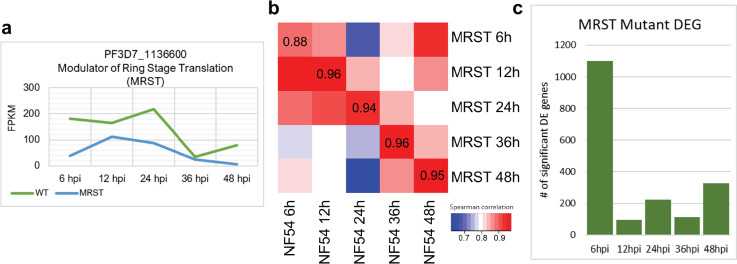
MRST disruption alters the early ring stage gene expression. (**a**) MRST gene FPKM expression analysis in NF54 and the mutant shows significant downregulation of MRST at 6 hpi (*P*-value: 0.00095). (**b**) Analysis of gene expression correlation of each harvested timepoint (6 hpi, 12 hpi, 24 hpi, 36 hpi, 48 hpi) between NF54 and MRST mutant. The lowest correlation between matched timepoint is seen at 6 hpi, with a Spearman correlation value of 0.88. Spearman correlation values and heatmap generated in R. (**c**) The number of significantly differentially expressed genes (DEGs) per timepoint in the MRST mutant was analyzed and plotted via bar graph. The highest number of DEGs were observed at 6 hpi, corresponding to the timepoint of MRST gene dysregulation, along with the identical timepoint of the lowest correlation between NF54 and mutant.

To determine the molecular pathways associated with the ART-S phenotypic changes in the MRST mutant, we investigated the transcriptomic differences between the mutant and NF54 WT during the IDC. Mutant and NF54 parent clone samples were harvested at 6 hours post-merozoite invasion (hpi) (early ring), 12 hpi (ring), 24 hpi (early trophozoite), 36 hpi (late trophozoite), and 48 hpi (schizont) for subsequent RNA sequencing and analysis (Data Set S1 Tab 4 and 5). Normalization, calculation of fold change expression, and statistical assessment of differential gene expression in the mutant were performed via DEseq2 (version 1.34.0). Significant downregulation of the MRST gene in the mutant compared to the NF54 parent occurred at 6 hpi and 48 hpi (6 hpi FC value: −2.342, 6 hpi *P*-value: 0.00095; 48 hpi FC value: −2.939; 48 hpi *P*-value: 0.00014) (Fig. 2a). We observed high transcriptional concordance between the MRST mutant and NF54 at the same IDC stages (Spearman correlation: 6 hours: 0.88; 12 hours: 0.96; 24 hours: 0.94; 36 hours: 0.96; 48 hours: 0.95) (Fig. 2b; Fig. S2; Data Set S1 Tab 6). A relatively moderate correlation was shown at 6 hours (Spearman correlation: 6 hours: 0.88), corresponding to the highest number of DEGs in the mutant (6-hour DEGs: 493 upregulated, 608 downregulated), compared to the other IDC stages at 12 hours (95 total DEGs), 24 hours (223 total DEGs), 36 hours (111 total DEGs), and 48 hours (328 total DEGs) (Fig. 2c).

We also counted the proportion of different IDC stages during parasite development via microscopy for our RNAseq samples, showing no significant difference in RNAseq samples stages and demonstrating the stage alignment between the MRST mutant and NF54 (Fisher’s exact test, *P*-value > 0.05) (Fig. S3; Data Set S1 Tab 7). Additionally, the transcriptional profiles in our study are highly consistent with the previously published NF54 and K13 *piggyBac* mutant transcriptome data (12). High correlation values were observed at identical timepoints between the NF54 parasites in the different studies (Spearman correlation: 6 hours: 0.87; 12 hours: 0.87; 24 hours: 0.85; 36 hours: 0.85; 48 hours: 0.87) (Fig. S4; Data Set S1 Tab 8) (12). Furthermore, the transcriptional correlation of housekeeping pathways (protein biosynthesis, DNA replication, transcriptional machinery, ribosome structure, fatty acid synthesis, redox metabolism, and genes coding for proteasome-related pathways) further demonstrates the life cycle consistency and supports metabolic similarity between NF54 and MRST mutant (Fig. S5; Data Set S1 Tab 9 and 10).

### Gene ontology enrichment of MRST mutant early ring stage expression reveals dysregulation in RNA metabolic processes

**Fig 3:**
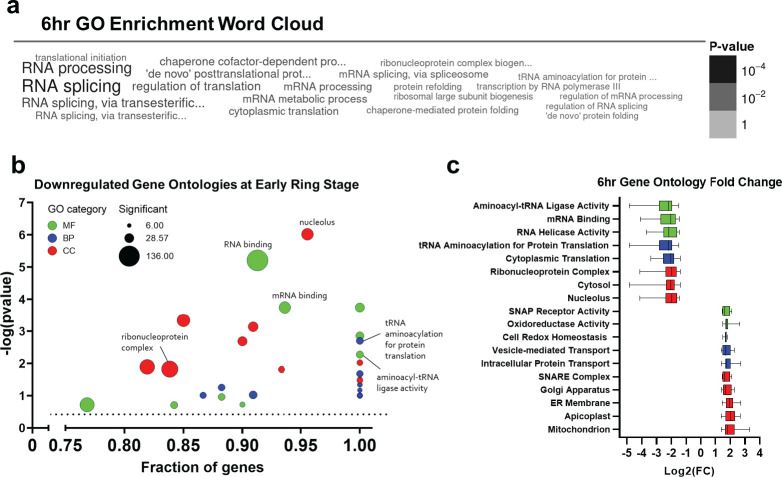
Gene ontology (GO) enrichment of 6 hpi expression in the MRST mutant. (**a**) GO enrichment of significantly dysregulated genes at 6 hpi in the mutant as shown in word cloud plot. Word color and size correspond to the *P*-value, and word cloud plot is generated via PlasmoDB GO enrichment tool. (**b**) In addition to PlasmoDB database GO enrichment, 6 hpi expression in the mutant was subjected to GO enrichment via the pfGO package in R. Downregulated GO terms were highly enriched in translational metabolism terms such as mRNA binding, ribonucleoprotein complex, and tRNA aminoacylation for protein translation. GO terms are plotted via bubble plot, with the x-axis corresponding to the fraction of annotated genes present in significant DEGs, and the y-axis corresponding to −log(*P*-value) of each term. Bubble size corresponds to the number of significant DEGs associated with the term in the mutant, and color corresponds to the GO category (green: molecular function, blue: biological process, red: cellular compartment). (**c**) Log(foldchange) plots of genes associated with select upregulated and downregulated GO terms at 6 hpi in the mutant. Plot is generated in GraphPad Prism.

We next interrogated the biological pathway alterations behind the high number of DEGs at 6 hpi between the MRST mutant and NF54. We performed GO enrichment analysis, via the PlasmoDB GO enrichment tool, for the dysregulated genes at 6 hpi (Fig. 3a), highlighting pathways such as RNA processing, RNA splicing, translational initiation, tRNA aminoacylation for protein translation, and ribonucleoprotein complex biogenesis dysregulation (*P*-value cutoff < 0.05) (Data Set S1 Tab 11). To further support these findings, additional GO enrichment analysis via the pfGO enrichment package in R was also performed on 6 hpi DEGs of the mutant (41, 42). Upregulated GO terms included terms associated with intracellular protein transport (intracellular protein transport *P*-value: 0.00054, vesicle-mediated transport *P*-value: 0.02788) and terms associated with the mitochondrial cellular compartment (*P*-value: 4.80E-07) (Data Set S1 Tab 12), while downregulated GO terms were significantly enriched in genes related to RNA binding (*P*-value: 6.20E-06), mRNA binding (*P*-value: 0.00018), ribonucleoprotein complex (*P*-value: 0.0151), and tRNA aminoacylation for protein translation (*P*-value: 0.002) (Fig. 3b; Data Set S1 Tab 12). Fold change expression values of genes associated with these top upregulated and downregulated GO terms are shown in Fig. 3c (Data Set S1 Tab 5 and 13).

### Protein biosynthesis pathways are significantly dysregulated at the early ring stage in the MRST mutant

**Fig 4:**
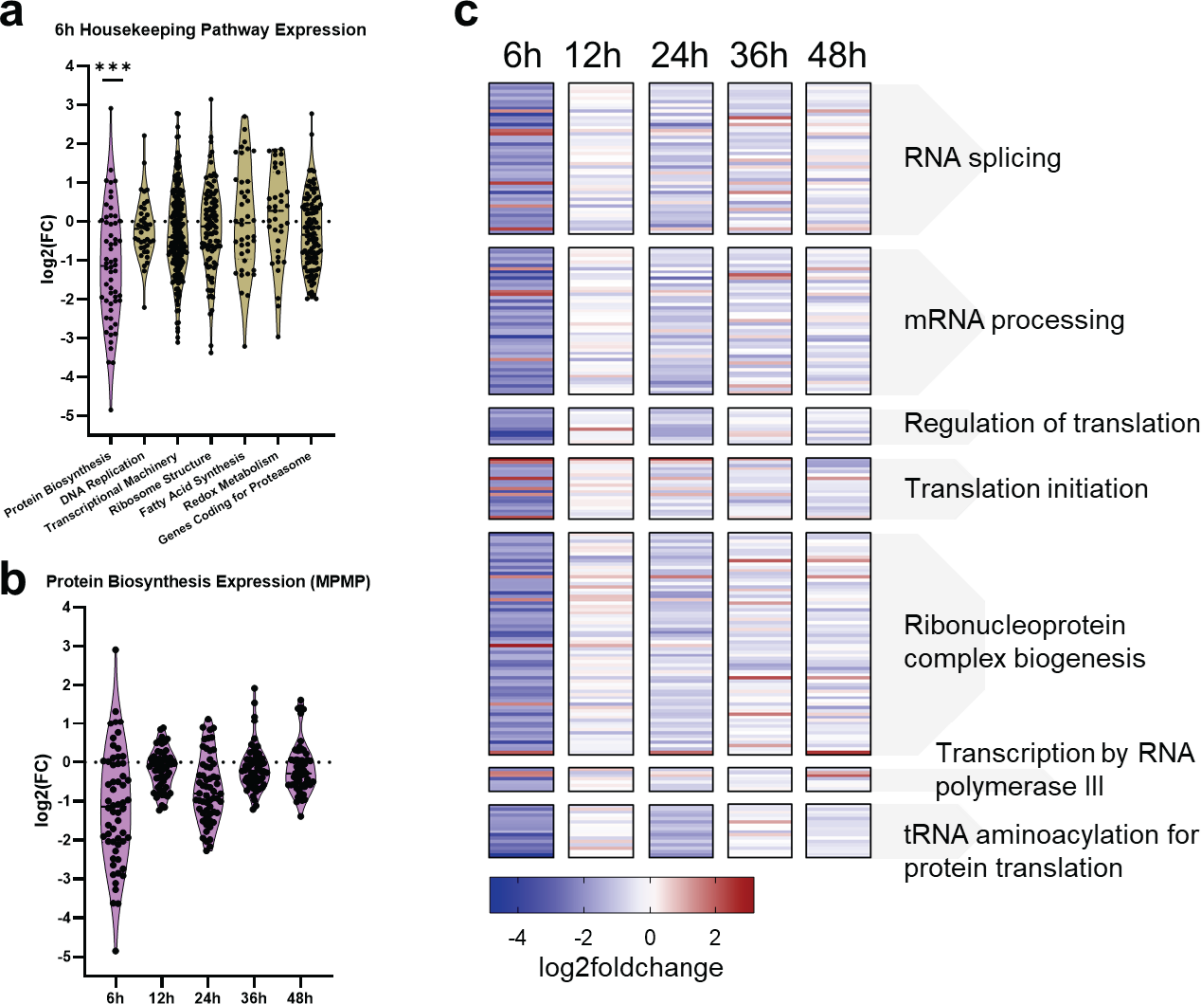
Protein biosynthesis–related pathways are significantly dysregulated at 6 hpi in the mutant. (**a**) Housekeeping pathway expression at 6 hpi was analyzed to determine the significance of translation-associated GO term dysregulation, with gene lists obtained from Malaria Parasite Metabolic Pathways database for protein biosynthesis, DNA replication, transcriptional machinery, ribosome structure, fatty acid synthesis, redox metabolism, and genes coding for the proteasome. Widespread downregulation was only observed for genes associated with protein biosynthesis. (**b**) Analysis of fold change expression of these same protein biosynthesis–related genes in the MRST mutant show downregulation at only 6 hpi, compared to the other timepoints tested. (**c**) Log2(foldchange) across all sampled timepoints was analyzed for gene sets corresponding to the various translation-associated GO terms as determined by pfGO analysis in R and displayed via heatmap. The majority of these translation-associated genes and pathways are solely downregulated at 6 hpi. Graph and statistics are generated in GraphPad Prism.

Using the Malaria Parasite Metabolic Pathways database (https://mpmp.huji.ac.il/maps/all), we chose to investigate the mutant gene expression of a select number of established housekeeping pathways in *P. falciparum* to identify the significance of dysregulated RNA processing and translation-related pathways at 6 hpi (21, 43 - 45). Gene sets were obtained corresponding to the following housekeeping pathways: protein biosynthesis, DNA replication, transcriptional machinery, ribosome structure, fatty acid synthesis, redox metabolism, and genes coding for the proteasome (Data Set S1 Tab 9). High correlations of these housekeeping pathways were seen between NF54 and the MRST mutant at identical timepoints (Fig. S5; Data Set S1 Tab 10). Additionally, we analyzed fold change expression values of genes corresponding to each pathway at 6 hours, with downregulation of protein biosynthesis–related genes seen compared to the other housekeeping pathways (Fig. 4a; Data Set S1 Tab 9). Analysis of fold change expression of the same protein biosynthesis–related genes in the mutant shows widespread dysregulation only at 6 hours, with no widespread change in gene expression at 12 hours, 24 hours, 36 hours, and 48 hours (Fig. 4b; Data Set S1 Tab 14).

To further validate significantly enriched gene ontologies at 6 hours in the mutant, as identified by PlasmoDB GO Enrichment analysis (Fig. 3a; Data Set S1 Tab 11), we analyzed fold change expression of genes associated with select 6- hour GO terms. As shown in Fig. 4c, we highlight the following pathways that were dysregulated at 6 hours: RNA splicing (*P*-value: 2.83E-05), mRNA processing (*P*-value: 0.00124), regulation of translation (*P*-value: 0.00062), translation initiation (*P*-value: 0.00281), ribonucleoprotein complex biogenesis (*P*-value: 0.00304), transcription by RNA polymerase III (*P*-value: 0.00419), and tRNA aminoacylation for protein translation (*P*-value: 0.00437). Fold change expression of genes in these pathways show dysregulation at 6 hours with a lack of gene dysregulation at the other harvested timepoints (Data Set S1 Tab 15). Because we observed significant dysregulation of the MRST gene also at 6 hours (*P*-value: 0.00095) (Fig. 2a) and identified dysregulation of translation associated genes at only 6 hpi in the mutant (Fig. 4c), this points to a potential link between MRST and translation gene dysregulation.

### MRST disruption leads to the dysregulation of ribosome biogenesis pathways causing downstream effects on translation

**Fig 5:**
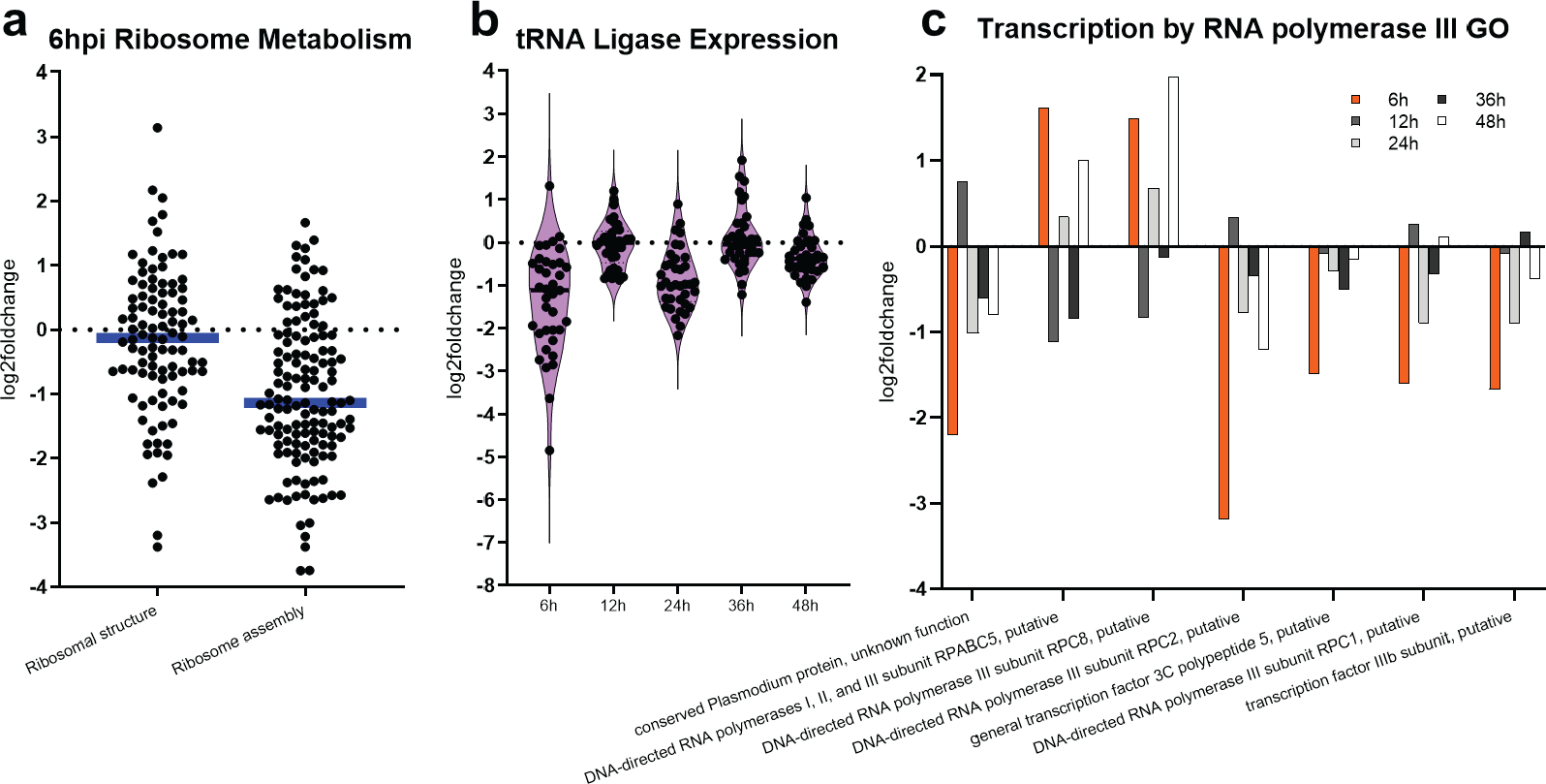
MRST disruption leads to the dysregulation of ribosome biogenesis pathways, causing downstream effects on translation expression. (**a**) Due to the bioinformatically predicted homology of the MRST protein to the midasin protein linker domain and disordered regions, ribosome metabolism pathway expression was analyzed at 6 hpi. While only 13 out of 97 genes (~13%) related to ribosome structure were downregulated at 6 hpi, 51 out of 133 genes (~38%) of the ribosome assembly pathway were downregulated, primarily pointing to the dysregulation of ribosomal assembly/maturation, and not the ribosome subunits themselves, being affected by MRST disruption. (**b**) Several tRNA ligases were differentially expressed at 6 hpi, with the majority lacking significant dysregulation at the other harvested timepoints in the mutant (*P*-value > 0.05). (**c**) Seven genes associated with RNA polymerase III activity (PF3D7_0609900, PF3D7_0708100, PF3D7_1104700, PF3D7_1206600, PF3D7_1210400, PF3D7_1329000, PF3D7_1449300) were dysregulated at 6 hpi compared to the other timepoints. Graphs and statistics are generated in GraphPad Prism.

MRST contains partial predicted similarity to the linker and disordered domains of the midasin domain of the AAA+ ATPase family, involved in the maturation and nuclear export of ribosome subunits (Fig. 1a). To further investigate the link between this predicted domain and the MRST mutant transcriptome, we chose to analyze differential expression of gene lists obtained from the MPMP database for ribosomal subunit genes and for genes coding for components involved in ribosomal assembly at 6 hpi. The majority of ribosomal subunit genes (79 out of 97 genes) lacked significant dysregulation at 6 hpi (*P*-value > 0.05), with only 13 out of 97 genes (~13%) being significantly downregulated (Fig. 5a; Data Set S1 Tab 16). In comparison, we observed more significant downregulation (*P*-value cutoff < 0.05) of genes coding for components involved in ribosome assembly (51 out of 133 genes, ~38%), including ATP-dependent RNA helicases, eukaryotic translation initiation factors, ribosome biogenesis proteins, and small nucleolar RNA-associated proteins (Fig. 5a; Data Set S1 Tab 16). This points to the initial assembly of ribosomes, instead of the ribosomal subunits themselves, as the primary dysregulated process of ribosomal maturation due to the MRST disruption.

We have shown that several protein biosynthesis pathways other than ribosome biogenesis are downregulated at 6 hpi in the mutant, including translation initiation, mRNA processing, transcription by RNA polymerase III, and tRNA aminoacylation (Fig. 4c). We identified 16 tRNA ligases, the majority of which (12 out of 16) are considered essential for asexual blood stage development (Fig. S7A), that have significant downregulation solely at the early ring stages (*P*-value cutoff < 0.05) (Fig. 5b; Fig. S7b and c; Data Set S1 Tab 15) (46). Additionally, several genes associated with RNA polymerase III, which transcribes DNA known to synthesize tRNA, were significantly dysregulated at 6 hpi including DNA-directed RNA polymerases I, II, and III subunit RPABC5 (PF3D7_0708100), DNA-directed RNA polymerase III subunit RPC8 (PF3D7_1104700), DNA-directed RNA polymerase III subunit RPC2 (PF3D7_1206600), and DNA-directed RNA polymerase III subunit RPC1 (PF3D7_1329000) (6 hpi *P*-value cutoff < 0.05) (Fig. 5c; Data Set S1 Tab 15) (47). Additionally, MAF1 (PF3D7_0416500), a negative regulator of RNA polymerase III, is not dysregulated at any stage of development in the MRST mutant, pointing to downstream effects associated with MRST dysregulation on ribosome assembly and other regulatory pathways of translation (47).

## DISCUSSION

With the urgent and potentially disastrous issue of artemisinin resistance and ACT failure in Asia and Africa, combined with the various molecular mechanisms of *P. falciparum* resistance, it is imperative to identify and characterize novel genes that can alter the artemisinin stress response. GWAS and TWAS have made substantial gains in detecting mutations associated with delayed parasite clearance in the field, along with characterizing transcriptional signatures linked to artemisinin resistance and response (15 - 18 - 25). However, while these studies focus on the large-scale analysis of field isolates and data sets to identify shared molecular mechanisms, they lack the comprehensive metabolic and transcriptomic characterization of unique individual genes in *P. falciparum*, many of which lack functional annotation, that can alter artemisinin response (17, 25, 46). Therefore, in this study we have transcriptionally characterized the *piggyBac* transposon mutant of the novel, unannotated gene PF3D7_1136600 (conserved *Plasmodium* gene, unknown function) that we have termed a modulator of ring stage translation (MRST) based on its bioinformatically predicted domains and associated transcriptional profile of the mutant.

Our study has several key findings and observations. First, we observed that *piggyBac* disruption in the 3′ untranslated region of the MRST gene led to the downregulation of expression at 6 hpi. Our data point to the significant dysregulation of MRST (*P*-value: 0.000953) as a factor in the differential gene expression seen at 6 hpi in the mutant. Genes related to mRNA binding, translation initiation, and ribosome assembly pathways were dysregulated at 6 hpi. Additionally, we identified the downregulation of seven genes associated with RNA polymerase III activity, along with the downregulation of 16 tRNA ligases. tRNA ligases are highly conserved housekeeping genes of the *P. falciparum* protein synthesis pathway, known for the charging of tRNAs for protein translation, and the majority are classified as essential for asexual stage development (46). The inhibition of tRNA ligase activity leads to an accumulation of uncharged tRNAs, associated with decreased protein synthesis and cell death, making tRNA ligases potent and widely studied antimicrobial drug targets (48 - 50). Additionally, dysregulation of RNA polymerase III proteins, also essential for asexual development, notably impacts tRNA ligase expression, ribosome biogenesis, RNA processing, and protein transport (47). Overall, we observe a multi-pronged dysregulation of protein biosynthesis regulatory pathways at the early ring stage that we suspect plays a role in the artemisinin-sensitive phenotype of the MRST mutant.

MRST is currently annotated as a conserved *Plasmodium* gene of unknown function (PF3D7_1136600); however, MRST was bioinformatically predicted to have putative partial similarity to the linker and disordered regions of the midasin protein, involved in the nuclear export and maturation of ribosomes (36). Supporting this is the identification of a predicted nuclear localization signal in MRST and the observed dysregulation in translational gene expression at 6 hpi, the same timepoint of MRST downregulation. While the majority of ribosomal subunit genes lacked significant dysregulation at 6 hpi, genes involved in the regulation of assembly of the ribosome are downregulated. Considering the annotated role of the midasin domain in ribosome nuclear export and maturation and the timepoints of MRST gene, translation pathways, and ribosome assembly dysregulation, our finding suggest that MRST may be mildly associated with ribosome maturation processes at the early ring stage. As our bioinformatic characterization of the MRST protein was preliminary to identify putative domains and characteristics to elucidate gene function, it will be important to perform additional analysis to determine how the moderate similarity with midasin is associated with MRST gene function.

Our predicted model is that MRST may be responsible for the early regulation of ribosome maturation processes, via moderate similarity to the midasin protein, at the early ring stage and, due to *piggyBac* transposon-mediated disruption, downregulation of MRST activity negatively impacts downstream effects on protein translation and the ability to respond to artemisinin-mediated stress at the ring stage. While we observed significant dysregulation of translational, tRNA ligase, and ribosomal genes, we did not observe significant impacts on cell cycle progression in the mutant compared to NF54 (Fig. 1e). Under normal growth, translation increases as the parasite matures, notably ramping up at the trophozoite/schizont border compared to the ring stage parasites (19, 51). Protein biosynthesis is already considerably low at the ring stage compared to other stages and, under normal conditions that the parasite samples in this study were subjected to, dysregulation may not affect parasite growth (19). However, as the ring stage is a critical timepoint for ART phenotypic response, dysregulation of translational activity may lead to decreased protein synthesis capacity under ART exposure, having lasting effects on later stages of parasite development.

MRST is considered essential for the asexual development and combined with the putative domains and transcriptome of the MRST mutant, supports MRST as an essential modulator for the ring stage translation. Due to the non-mutability of MRST during asexual stage, future studies will need to involve targeted mutagenesis of specific regions of the gene, such as a knockout of the predicted midasin homology regions or nuclear localization signal sites, or epitope tagging of MRST to confirm protein localization throughout the IDC with and without DHA stress. Lastly, it will be important to measure protein production throughout the life cycle, achievable through liquid chromatography with tandem mass spectrometry, to confirm MRST’s role in altering protein translation and activity. Combined, these future studies will give us more clarification on the function of MRST and the essential role this gene may play in ribosome metabolism and translation in *P. falciparum*.

In conclusion, we have performed an initial characterization of the novel PF3D7_1136600 gene, which we have termed MRST, via the analysis of the transcriptome of its *piggyBac* mutant, predicting that this gene plays a role in ribosome assembly and translational activity during the ring stage. With many genes of the *P. falciparum* genome lacking functional annotation, our putative annotation of the MRST gene contributes to the critical goal of characterizing novel drug–gene interactions. There is a continuing need to comprehensively characterize genetic factors that can alter artemisinin response in *P. falciparum* to understand mechanisms of resistance along with identifying novel drug targets. With this being the first characterization of how MRST can alter the transcriptome of *P. falciparum*, we hope our findings can provide an additional step into the characterization of this gene and other novel molecular interactions between *P. falciparum* and artemisinin.

## MATERIALS AND METHODS

### Parasite culturing methods

Standard *in vitro P. falciparum* culturing protocols were used to freeze, thaw, and maintain culture (52). Parasite cultures were maintained in human O+ red blood cells (RBCs) (obtained from commercial supplier Interstate Blood Bank, Inc.) at 4% hematocrit in culture media containing RPMI 1640 supplemented with HEPES, hypoxanthine, sodium bicarbonate, Albumax II, and gentamicin. A 37°C mixed gas incubator was used with O_2_, CO_2_, and N_2_. *piggyBac* mutants were created in the wild-type (WT) isogenic NF54 parent (including a WT K13), which is the parent line of the 3D7 clone, and validated via thermal asymmetric interlaced (TAIL)-PCR (32). Asexual phenotypic growth of mutant clones was first characterized (53). The NF54 line, containing no known K13 ART-R mutations and sensitive to antimalarials, was re-cloned and sequenced to serve as the reference genome for insertion mapping (46, 54). The MRST mutant was first described as *piggyBac* mutant 52 (PB52) in a previous study via TAIL-PCR (32). *piggyBac* mutants, including the MRST mutant and the previously characterized K13 *piggyBac* mutant PB58 (12), contain a NF54 genetic background except for a single *piggyBac* transposon insertion (an MRST 3′ untranslated region [UTR] insertion and K13 5′ UTR insertion for PB52 and PB58, respectively).

### Bioinformatic characterization of MRST protein

The protein sequence of MRST was input into the National Center for Biotechnology Information (NCBI) Protein Blast database (https://blast.ncbi.nlm.nih.gov/Blast.cgi) to determine conserved protein sequences and putative domains (accessed July 2022) using the MRST protein sequence available on the PlasmoDB database (35, 55). ExPasy Prosite Database was used (August 2022) to identify putative localization signals, amino acid-rich regions, and other bioinformatically determined protein characteristics (37).

### SYBR Green I malaria drug sensitivity assay

The standardized SYBR Green I drug sensitivity assay was performed to determine altered sensitivity to ART drugs and other antimalarials (38, 39). Thawed parasite clones were allowed to reach ~2% parasitemia in culture before being synchronized with 10% sorbitol at the majority ring stage. Cultures were sorbitol synchronized at the next cycle of ring stage and allowed to recover for another 48-hour life cycle before starting the assay. A 1 mg/mL stock solution of DHA was diluted to the appropriate mother plate starting concentration, then diluted two-fold in 96-well Greiner black bottom well plates, leaving the last column for positive and negative drug controls. Ten microliters of mother plate drugs were added to the 96-well daughter plate containing 90 μL of parasite culture (2% hematocrit, 0.5% parasitemia) and incubated in a mixed gas incubator for 72 hours. Plates were then removed, frozen at −20°C for 24 hours, removed, and allowed to thaw to room temperature (RT), and 100 μL of 4× SYBR Green I dye solution (from an initial 10,000× SYBR Green stock) in 1:1 lysis buffer:water solution was added to each plate well. For the lysis buffer solution, dissolve 15.8 g Tris-HCl in 964-mL culture water (adjust pH to 7.5). Add 20 mL of 0.5M EDTA, 160 mg of saponin (0.016% wt/vol), and 16 mL of Triton X-100 (1.6% vol/vol) before mixing and sterilizing via vacuum filtration. After incubating plates at RT for 1 hour, SYBR Green fluorescence was read using the ClarioStar plate reader and analyzed via Excel and GraphPad Prism. IC50 values were determined by non-linear regression (sigmoidal dose–response/variable slope equation) of plotted normalized % inhibition values versus log(drug concentrations). Statistical analysis of IC50 values performed via Mann–Whitney test in GraphPad Prism. The assay was performed with six biological replicates.

### Ring stage survival assay

Ring stage survival assay (RSA) was conducted for NF54 and MRST mutant to validate mutant artemisinin sensitivity (40). Parasite clones were thawed and maintained in culture until reaching 2%–5% majority ring stage for sorbitol synchronization. Sorbitol synchronization was again performed 30–48 hours later depending on parasite development and stages at the time of sorbitol treatment. After 30 hours of culture (majority late schizont stages), cultures were subjected to Percoll synchronization for late-stage schizont enrichment (70%:40% gradient). Thin smear slides were taken, and parasites returned to culture with the appropriate volume of complete media and uninfected RBC added. The culture was allowed to grow as normal in a mixed gas incubator for exactly 3 hours. Thin smear slides were then taken to evaluate the proportion of ring stages (>0.5%), cultures were incubated with sorbitol for 10 minutes, parasitemia was made to 0.5%–1% with a 2% hematocrit, and thin smear slides were taken again as initial slides “INI.”

Previously prepared DHA test solution (700 nM concentration) and dimethyl sulfoxide (DMSO) control solution were added to a 24-well culture plate under the following parameters: 100 μL of DMSO solution to the “non-exposed” wells and 100 μL of DHA solution to the “DHA-exposed” well. Infected RBC suspension of 900 μL was added to each well, with the 24-well plate being maintained in a mixed gas incubator for exactly 6 hours. Following the 6 hours, the contents of each well were washed with RPMI. The contents of each well were added to 15-mL centrifuge tubes, pelleted at 1,750 rpm for 3 minutes, the supernatant discarded, and 10 mL of RPMI added. Cultures were again pelleted, the supernatant discarded, and washed again with 10 mL of RPMI. Cultures were pelleted for a final time before the supernatant was discarded, 1 mL of complete culture media added, and returned to culture at 2% hematocrit in a new 24-well plate. Thin smear slides were taken for each well, and cultures were maintained in a mixed gas incubator for 66 hours. Following incubation, thin smear slides were taken for each well for microscopic analysis.

Slides were classified as “INI” smear to define the initial parasitemia, “NE” smear to define the non‐exposed parasitemia at 72 hours, and “DHA” smear to define the DHA‐exposed parasitemia at 72 hours. The number of infected RBCs containing viable parasites were counted (in a total of 10,000 RBCs estimated by the number of RBCs per field). The percent survival (% survival) values in NE and DHA slides are calculated using the following equation:


$$Growth\;rate=\frac{NE}{INI}$$

$$Percentage\;survival\;\;\;(\%)=\frac{DHA}{NE}\times 100$$

Percentage survival values are interpretable if the growth rate is ≥1.5 for the *in vitro* RSA.

#### Intraerythrocytic development cycle growth assay

The MRST mutant was analyzed for altered IDC progression under DHA exposure compared to NF54. Clones were thawed via standard protocols and synchronized at the same timepoint using 10% sorbitol for ring stage enrichment. After reaching ~2% majority ring, clones were sorbitol synchronized three times every 48 hours and allowed an additional 48 hours for recovery to obtain highly synchronized rings. When the NF54 parent clone reached 0 hpi (about half schizonts and half rings) (56), microscopy slides were taken for each culture every 4 hours for 48 consecutive hours, with culture media and fresh RBCs being replenished when needed. For the DHA drug pressure group, DHA at NF54 IC50 (concentration was added to each culture starting at 0 hpi, pulsed for 6 hours (with slides being taken at 0 hpi and 4 hpi) after which cultures were washed with RPMI twice, and returned to culture. Slides were taken post-washing of RBC cultures (~6 hpi) and continued to be taken every 4 hpi (8 hpi, 12 hpi, 16 hpi, etc.). Following 48 hours, all assay slides were counted to determine parasitemia and percentages of ring, trophozoite, and schizont per slide (dividing the total number of rings, trophozoite, or schizonts counted by the total number of parasites counted, multiplied by 100 to get a percentage of each stage). Data were analyzed using Excel spreadsheet, GraphPad Prism, and R. Percentage ring, trophozoite, or schizont were plotted against the timepoint post-merozoite invasion per clone. GraphPad Prism 9.3.1 was used to generate graphs, and R was used to perform Fisher’s exact test of cell counts per mutant and per timepoint for drug and no drug treatment.

#### Whole-genome sequencing and analysis

The MRST mutant and NF54 WT parent clone were subjected to whole-genome sequencing (WGS) to confirm a lack of additional mutations and to confirm the insertion site sequence in the mutant. Clones were thawed via standard thawing protocols and maintained in a 20-mL culture at 4% hematocrit in complete RPMI media. Cultures were allowed to reach ~5% parasitemia of the majority of schizonts before being subjected to lysis and DNA extraction. Briefly, RBCs and parasitized RBCs in culture were pelleted at 1,750 rpm for 3 minutes in a 50-mL centrifuge tube. The supernatant was removed, and cells were resuspended in 20-mL 1× PBS (phosphate-buffered saline). One hundred microliters of 10% saponin was added to the solution to lyse RBCs, and the tube was inverted three to four times to mix. Crude lysed cells were then pelleted, and the supernatant was removed. Lysed cells were washed three times with 10 mL of 1× PBS and resuspended in 2 mL of 1× PBS before continuing with DNA extraction via the Qiagen QIAamp DNA Blood Mini Kit, with DNA concentration determined via Qubit 4 Fluorometer. The NEBNext Ultra II DNA Library Preparation Kit was used to prepare samples for sequencing, and samples were sequenced using NextSeq 550 at 300 cycles. The sequencing reads’ quality was assessed using FastQC (v0.11.5) and were trimmed to remove adapter and low-quality bases using Trimgalore (v0.4.4). Raw reads were mapped to the *P. falciparum* 3D7 reference genome (PlasmoDB version 58) using mapped reads from Burrows-Wheeler aligner supermaximal exact matches (sequence aligner for reads > 70 bp) (BWA-MEM ) (v0.7.17). The sequence alignment data were then post-processed using SAMtools (v1.3.1). The Integrative Genomics Viewer was used to visualize reads mapped at the *piggyBac* insertion site. Bedtools (version 2.28.0) was used for comparative analysis to evaluate the presence of the mutations, such as SNPs and indels.

#### RNA extraction and sequencing

Five timepoints during the 48-hour IDC were harvested for RNAseq analysis in this study: 6 hours post-invasion (hpi), 12 hpi, 24 hpi, 36 hpi, and 48 hpi. Tightly synchronous isogenic WT NF54 and MRST mutant were obtained via three rounds of sorbitol synchronization for ring stage isolation and one round of Percoll synchronization for schizont stage isolation, maintained in a 200-mL culture at 4% hematocrit and ~5% parasitemia. 0 hpi of the culture was estimated via microscopy and samples were harvested at the correct timepoints, with morphological stages analyzed similar to growth screen analysis via Excel, GraphPad Prism, and Fisher’s exact test of microscopy cell counts in R. The standard TRIzol extraction method was used to extract RNA from crude saponin–lysed samples. Cultures were transferred from culture flasks to conical tubes, centrifuged at 1,750 rpm for 3 minutes, and supernatant was removed. RBC pellets were resuspended in cold 1× PBS, and RBCs were lysed via saponin, followed by washing three times afterward with cold PBS. The pellet was resuspended in 1-mL TRIzol for every 50 mL of culture and stored at –80°C for RNA extraction. Before extraction, samples were allowed to reach RT, and 2 µL of glycogen was added. The samples were incubated at 4°C overnight, removed, and allowed to reach RT. After adding chloroform, the samples were vortexed vigorously and incubated at RT for 5 minutes, followed by centrifugation at 12,000× *g* at 4°C for 10 minutes, supernatant discarded, and 1 mL of 75% ethanol was added. This was again followed by centrifugation at 10,000× *g* for 5 minutes, the supernatant was discarded, and the RNA pellet was allowed to dry. RNA pellets were then dissolved in 20–50 µL of diethyl pyrocarbonate (DEPC)-treated water while being incubated at 55°C for 10 minutes. RNA was quantified using a Qubit fluorometer RNA HS Assay kit, and quality was determined via RNA ScreenTape Analysis. Using the Illumina TruSeq Stranded mRNA Library Prep Kit, mRNA was sequenced using Illumina Nextseq Mid-output X5 kit at 300 cycles.

#### Gene expression determination

Gene expression data were obtained using previously described protocols (12). Reads from RNA sequencing (RNAseq) were aligned with the *P. falciparum* 3D7 reference clone via the HISAT2 program (Hierarchical indexing for spliced alignment of transcripts). FeatureCounts read summarization program was used to obtain raw counts. The expression of each gene was normalized and calculated using Cuffnorm as fragments per kilobase per million mapped reads (FPKM).

#### DEseq2 analysis to determine gene dysregulation

Normalization, fold change (FC) calculation (MRST mutant/NF54), and statistical assessment of differential gene expression (DE) were performed via DEseq2 (version 1.34.0). A log2FC > 1.4 and false discovery rate (FDR)-corrected *P*-value < 0.05 classified genes are significantly upregulated in the mutant. log2FC < −1.4 and FDR-corrected *P*-value < 0.05 determined genes are significantly downregulated in the mutant. DEseq2 analysis performed in R.

#### Transcriptional alignment and correlation assessment

To determine the similarity in gene expression patterns between NF54 and the MRST mutant, FPKM gene expression values per timepoint were analyzed for expression correlation. Using R and cor.test function, FPKM values of genes for NF54 and mutant were correlated to determine Spearman’s rank correlation coefficient. To support that our samples are correctly transcriptionally aligned, NF54 and MRST mutant data sets from this study were compared to sequencing data from our previously reported K13 *piggyBac* mutant study from Gibbons et al. (12). Spearman’s rank correlation coefficient was determined between sample timepoints of both clones from both data sets as previously described in R. Genes were disregarded unless expression data were present in samples from both studies. Housekeeping pathways were then analyzed for correlated gene expression between NF54 and MRST clones in this study to determine transcriptional alignment at the metabolic pathway level. Gene lists corresponding to seven housekeeping pathways (protein biosynthesis, DNA replication, transcriptional machinery, ribosome structure, fatty acid synthesis, redox metabolism, and genes coding for proteasome) were obtained from the Malaria Parasite Metabolic Pathways database (https://mpmp.huji.ac.il/maps/all). FPKM values for each pathway for all timepoints sampled were analyzed for Spearman correlation between NF54 and MRST mutants in R. Correlation coefficients were plotted via heatmap.2 function in R in a heatmap.

### Gene ontology enrichment analysis

To analyze DEGs, gene ontology (GO) enrichment was performed for 6 hpi DEGs in the mutant. Significantly upregulated and downregulated gene IDs (upregulated > 1.4 log2FC, downregulated < −1.4 log2FC, significant *P*-value < 0.05) were input into the PlasmoDB database GO enrichment tool and analyzed for biological process of GO terms (55). GO terms were plotted with word cloud plot in PlasmoDB, with a 0.005 *P*-value cutoff to highlight the top significantly determined GO terms in the figure (55).

Additional GO enrichment assessment of 6 hpi expression in the mutant was performed by analyzing GO terms mapped to genes in a category of interest compared to a background of GO terms mapped to all other genes in the analysis using the R package pfGO (v 1.1) (41, 42). A GO-term database was created from the latest curated *P. falciparum* ontology available from PlasmoDB (accessed June 2022). GO terms are enriched in dysregulated gene categories in the mutant versus NF54 by timepoint and ontology, with “Up” and “Down” representing upregulation in the mutant or downregulation in the mutant compared to NF54, respectively. Enrichment was assessed via weighted Fisher’s/elim-hybrid *P*-value ≤ 0.05. Upregulated and downregulated GO terms were plotted in bubble plot, and/or box and whisker graphs were made in GraphPad Prism.

#### Metabolic pathway gene expression analysis

Select housekeeping pathways were chosen for gene expression analysis and gene expression correlation in the mutant. Gene lists corresponding to housekeeping pathways were obtained from the Malaria Parasite Metabolic Pathways database (https://mpmp.huji.ac.il/maps/all) and downloaded (June 2022) into Excel. Gene lists corresponding to each pathway were used to obtain log2FC (mutant/WT) expression values (generated via DEseq) in the mutant across the sampled timepoints. Violin plots of log2FC gene expression were generated and statistically analyzed in GraphPad Prism. Heatmaps of various protein biosynthesis pathways (obtained from pfGO GO enrichment analysis [41, 42]) was generated with log2FC values in GraphPad Prism. Significantly differentially expressed tRNA ligase gene dot plots and line graphs, along with RNA polymerase III–related genes bar graphs, were also generated and analyzed in GraphPad Prism.

## Data Availability

RNAseq data generated for NF54 and MRST mutants in this study have been deposited to the NCBI Gene Expression Omnibus (GEO) database with the accession number GSE223497. Processed RNAseq data are provided in Data Set S1 Tab 4 and Data Set S1 Tab 5. The IDC reference data can be obtained from Bozdech Z, Llinás M, Pulliam BL, Wong ED, Zhu J, DeRisi JL. 2003. The transcriptome of the intraerythrocytic developmental cycle of *Plasmodium falciparum*. PLoS Biol 1:e5. https://doi.org/10.1371/journal.pbio.0000005 WGS data for NF54 and MRST mutants in this study are available via the NCBI SRA with the BioProject ID PRJNA947639. The *Plasmodium falciparum* NF54 reference genome can be obtained from ftp://ftp.sanger.ac.uk/pub/project/pathogens/Plasmodium/falciparum/NF54/Assembly/V1_morphed/

## SUPPLEMENTAL MATERIAL

Fig S1 msphere.00152-23-s0001.pdf Growth screen.

Fig S2 msphere.00152-23-s0002.pdf RNAseq correlations.

Fig S3 msphere.00152-23-s0003.pdf RNAseq sample stage analysis.

Fig S4 msphere.00152-23-s0004.pdf Correlation of RNAseq samples.

Fig S5 msphere.00152-23-s0005.pdf Correlation to housekeeping pathways.

Fig S6 msphere.00152-23-s0006.pdf GO 6 hrs.

Fig S7 msphere.00152-23-s0007.pdf tRNA ligase expression.

Supplemental data set msphere.00152-23-s0008.xlsx Tables of quantitative data sets.

ASM does not own the copyrights to Supplemental Material that may be linked to, or accessed through, an article. The authors have granted ASM a non-exclusive, world-wide license to publish the Supplemental Material files. Please contact the corresponding author directly for reuse.
