# Supplementary material for: A novel Modulator of Ring Stage Translation (MRST) gene alters artemisinin sensitivity in Plasmodium falciparum
Source: mSphere. 2023 May 23;8(4):e00152-23. doi: 10.1128/msphere.00152-23 (PMC10449512; doi:10.1128/msphere.00152-23)
Supplement: Fig S1 — Growth screen. [file msphere.00152-23-s0001.pdf]

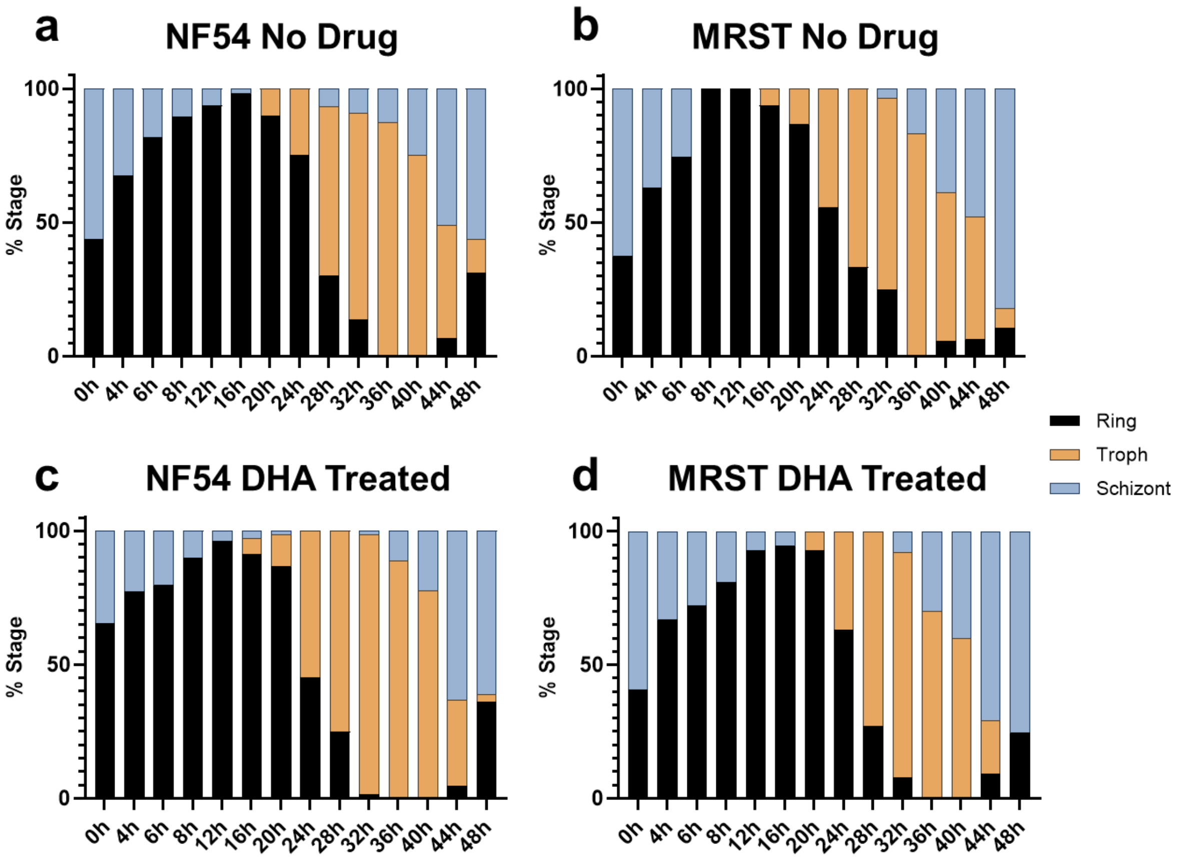
a
NF54 No Drug
b
MRST No Drug
c
NF54 DHA Treated
d
MRST DHA Treated
% Stage
100
50
0
0h
4h
6h
8h
12h
16h
20h
24h
28h
32h
36h
40h
44h
48h
Ring
Troph
Schizont

Supplementary Figure 1. Asexual cell cycle developmental stage analysis of DHA and non-DHA treated NF54 and MRST mutant clones. Synchronized early ring stage NF54 and MRST mutant clones were either allowed to grow without drug treatment (**a**, **b**) or subjected to a 6hr drug pulse of 600nM dihydroartemisinin (**c**, **d**), washed, and allowed to grow for 48hrs. Percentages of ring, trophozoite, and schizont stages of NF54 and MRST mutant were analyzed via microscopy every four hours, with statistical significance determined via Fisher's exact test of cell counts per timepoint (significant *p-value* < 0.05). No significant difference was found in the asexual cell cycle progression with and without a DHA drug pulse between NF54 and MRST mutant. Cell counts, stage percentages, and statistical analysis of no drug and drug treated clones are available in Data Set S1 Tab 3.
