# Supplementary material for: A novel Modulator of Ring Stage Translation (MRST) gene alters artemisinin sensitivity in Plasmodium falciparum
Source: mSphere. 2023 May 23;8(4):e00152-23. doi: 10.1128/msphere.00152-23 (PMC10449512; doi:10.1128/msphere.00152-23)
Supplement: Fig S2 — RNAseq correlations. [file msphere.00152-23-s0002.pdf]

Correlation of RNAseq Samples

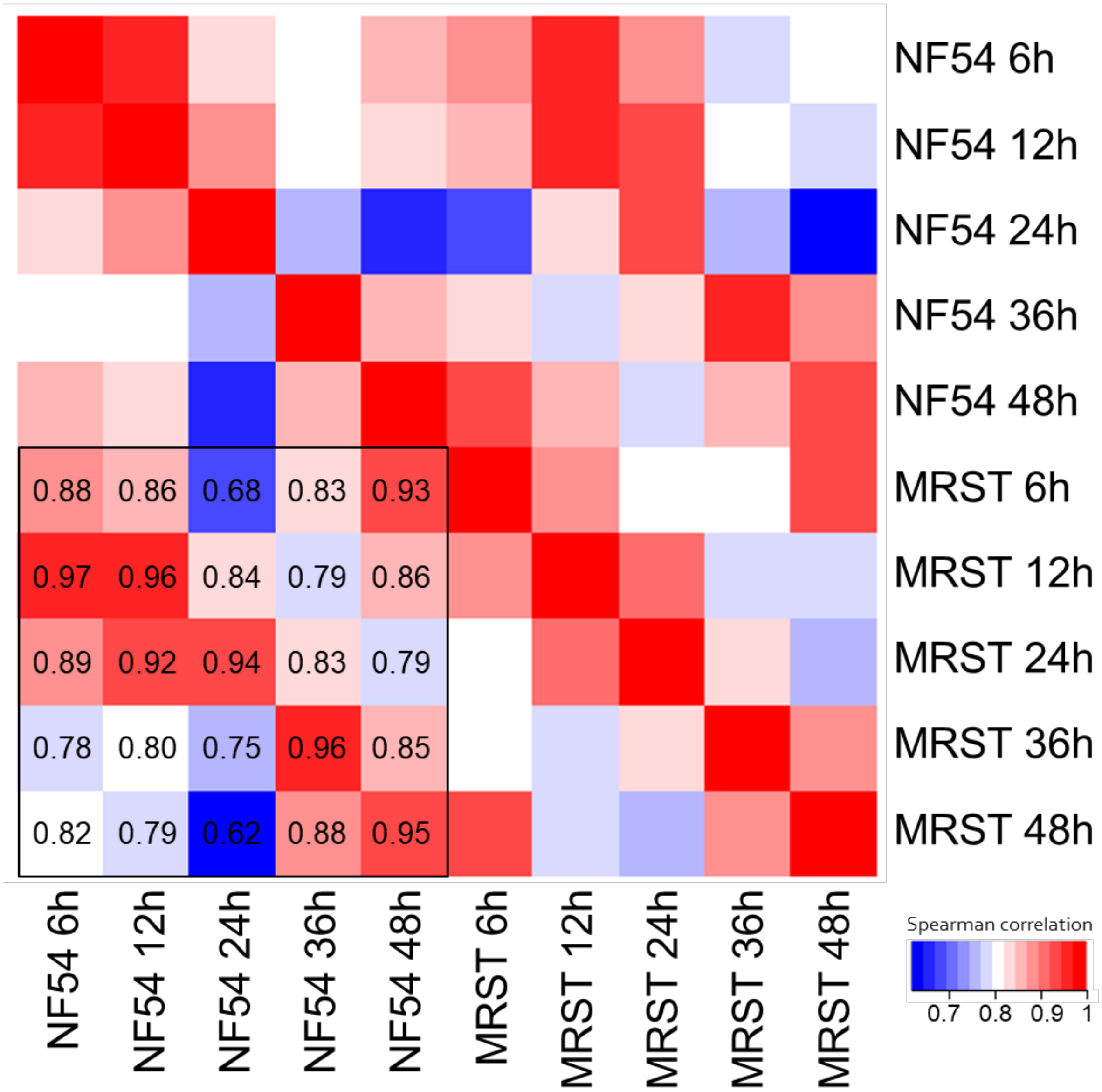

Supplementary Figure 2. Correlation of NF54 and MRST mutant expression profiles per timepoint. Via R, spearman correlation values between all NF54 and MRST mutant gene expression timepoints analyzed in this study were obtained and plotted via heatmap using the heatmap.2 function. Direct correlations between NF54 and MRST mutant are highlighted in the black box, showing transcriptional similarity between the two clones. All spearman correlation values are available in Data Set S1 Tab 6.
