# Supplementary material for: A novel Modulator of Ring Stage Translation (MRST) gene alters artemisinin sensitivity in Plasmodium falciparum
Source: mSphere. 2023 May 23;8(4):e00152-23. doi: 10.1128/msphere.00152-23 (PMC10449512; doi:10.1128/msphere.00152-23)
Supplement: Fig S3 — RNAseq sample stage analysis. [file msphere.00152-23-s0003.pdf]

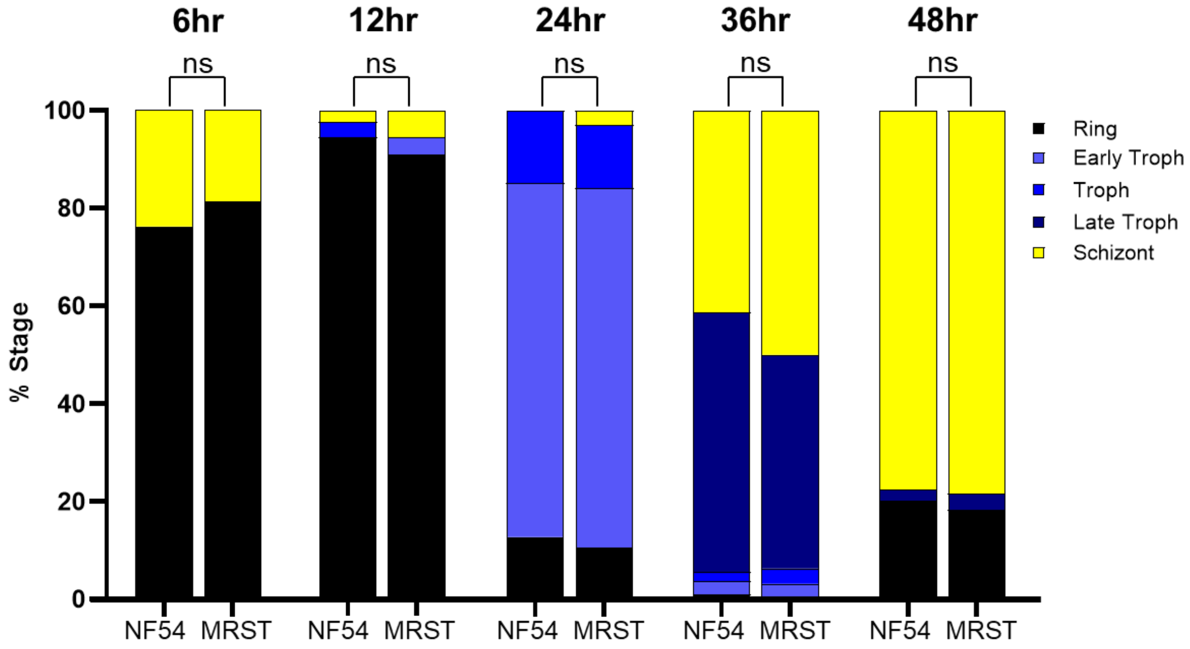
6hr
12hr
24hr
36hr
48hr
ns
ns
ns
ns
ns
% Stage
100
80
60
40
20
0
NF54
MRST
NF54
MRST
NF54
MRST
NF54
MRST
NF54
MRST
Ring
Early Troph
Troph
Late Troph
Schizont

Supplementary Figure 3. Cell cycle comparison between NF54 and MRST mutant RNAseq samples. To support transcriptional similarity, percentages of ring, early trophozoite, trophozoite, late trophozoite, and schizont stages were determined via microscopy for the RNAseq samples harvested for this study. Stage percentages per timepoint of NF54 and mutant were plotted via bar graph in GraphPad Prism, and statistical significance for each timepoint determined via Fisher's exact test (significant *p-value* < 0.05). No significant difference in cell cycle progression was observed between NF54 and MRST mutant RNAseq samples. Cell counts, stage percentages, and statistical analysis are available in Data Set S1 Tab 7.
