# Supplementary material for: A novel Modulator of Ring Stage Translation (MRST) gene alters artemisinin sensitivity in Plasmodium falciparum
Source: mSphere. 2023 May 23;8(4):e00152-23. doi: 10.1128/msphere.00152-23 (PMC10449512; doi:10.1128/msphere.00152-23)
Supplement: Fig S4 — Correlation of RNAseq samples. [file msphere.00152-23-s0004.pdf]

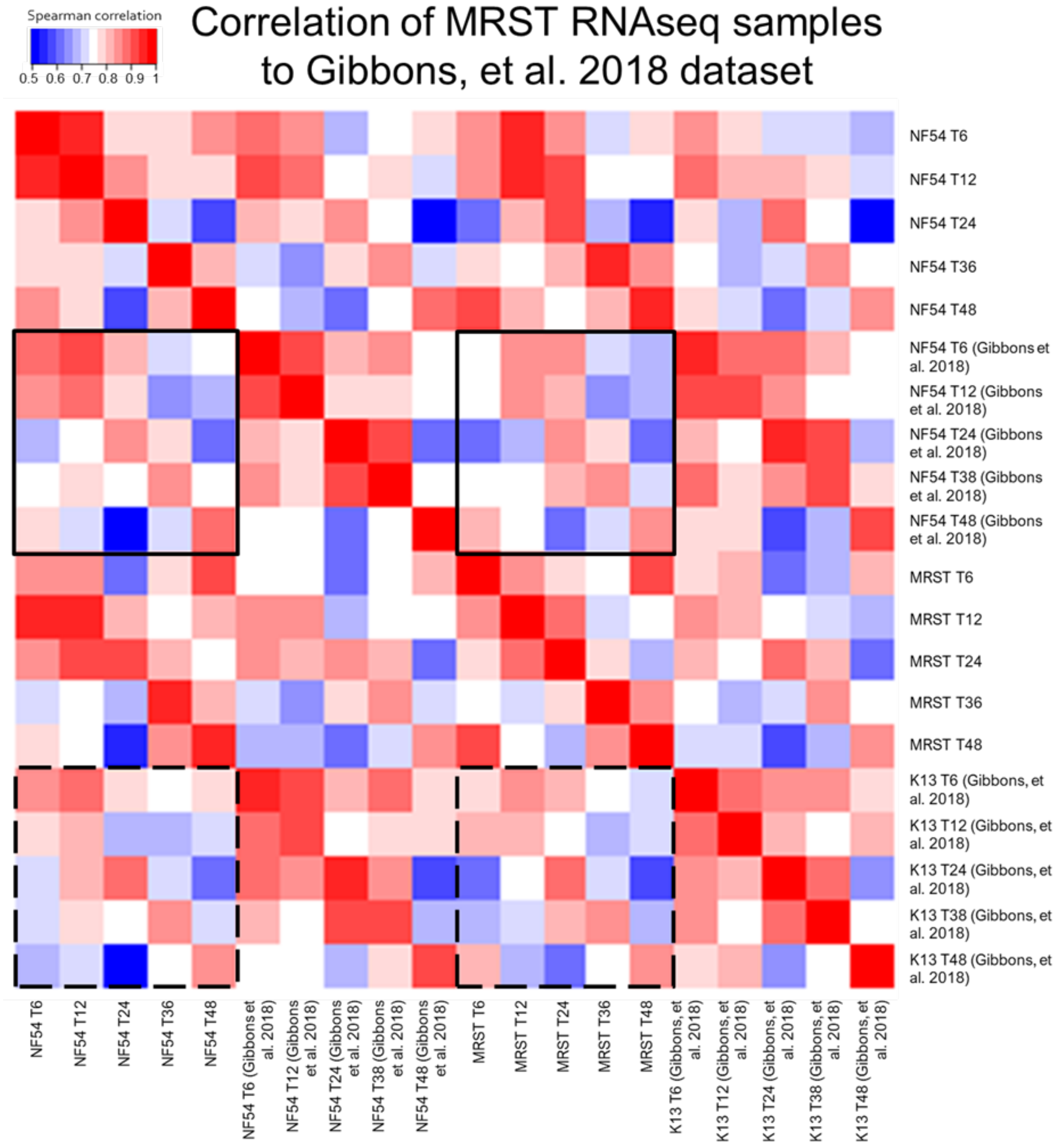
Correlation of MRST RNAseq samples to Gibbons, et al. 2018 dataset
Spearman correlation
0.5 0.6 0.7 0.8 0.9 1
NF54 T6
NF54 T12
NF54 T24
NF54 T36
NF54 T48
NF54 T6 (Gibbons et al. 2018)
NF54 T12 (Gibbons et al. 2018)
NF54 T24 (Gibbons et al. 2018)
NF54 T38 (Gibbons et al. 2018)
NF54 T48 (Gibbons et al. 2018)
MRST T6
MRST T12
MRST T24
MRST T36
MRST T48
K13 T6 (Gibbons, et al. 2018)
K13 T12 (Gibbons, et al. 2018)
K13 T24 (Gibbons, et al. 2018)
K13 T38 (Gibbons, et al. 2018)
K13 T48 (Gibbons, et al. 2018)

**Supplementary Figure 4**. NF54 and MRST mutant expression correlated to NF54 and K13 mutant expression profiles from Gibbons, et al. 2018. The NF54 and MRST mutant RNAseq samples from this study were analyzed in R for spearman correlation to the NF54 and K13 piggyBac mutant RNAseq samples previously published by Gibbons, et al. 2018. Heatmap.2 function in R was used to make a heatmap showing correlation between our samples and the published NF54 dataset (solid black boxes) and the published K13 mutant dataset (dashed black boxes). High correlation is seen between our RNAseq samples and published RNAseq samples, supporting correct transcriptional alignment. Spearman correlation values are available in Data Set S1 Tab 8.
