# Supplementary material for: A novel Modulator of Ring Stage Translation (MRST) gene alters artemisinin sensitivity in Plasmodium falciparum
Source: mSphere. 2023 May 23;8(4):e00152-23. doi: 10.1128/msphere.00152-23 (PMC10449512; doi:10.1128/msphere.00152-23)
Supplement: Fig S5 — Correlation to housekeeping pathways. [file msphere.00152-23-s0005.pdf]

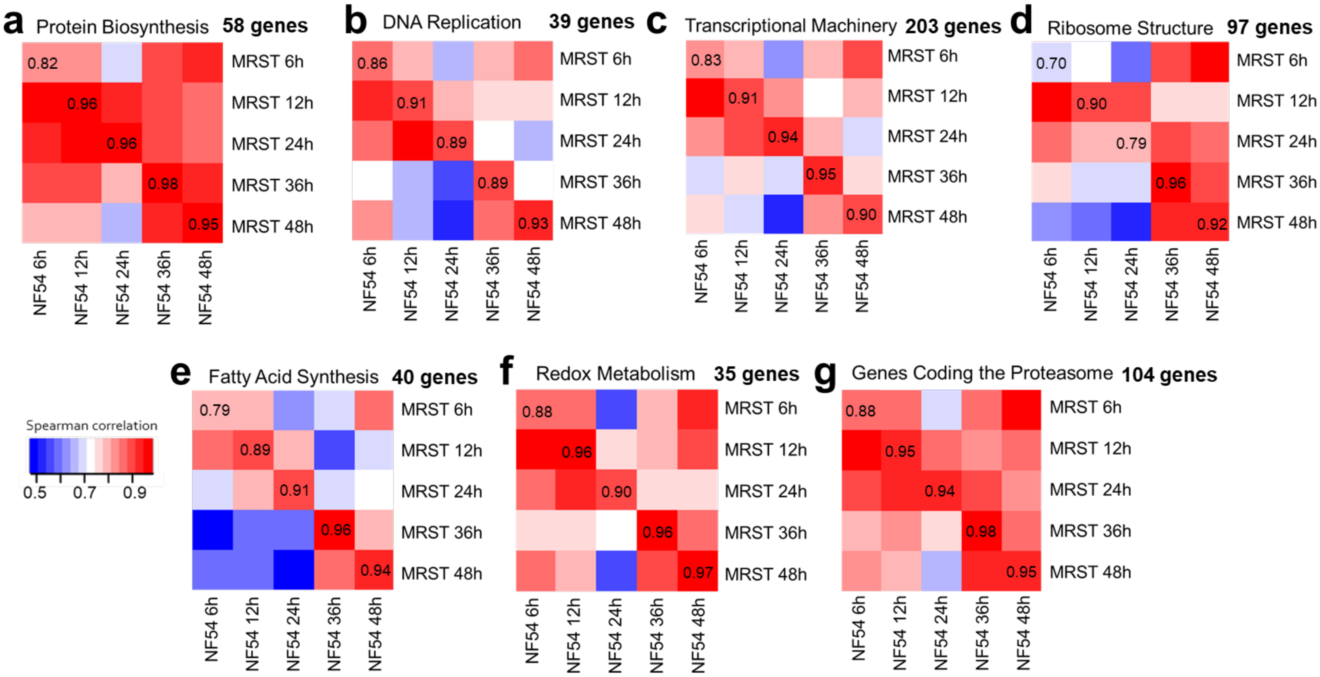
a Protein Biosynthesis 58 genes
b DNA Replication 39 genes
c Transcriptional Machinery 203 genes
d Ribosome Structure 97 genes
e Fatty Acid Synthesis 40 genes
f Redox Metabolism 35 genes
g Genes Coding the Proteasome 104 genes
Spearman correlation
0.5
0.7
0.9
MRST 6h
MRST 12h
MRST 24h
MRST 36h
MRST 48h
NF54 6h
NF54 12h
NF54 24h
NF54 36h
NF54 48h
0.82
0.96
0.96
0.98
0.95
0.86
0.91
0.89
0.89
0.93
0.83
0.91
0.94
0.95
0.90
0.70
0.90
0.79
0.96
0.92
0.79
0.89
0.91
0.96
0.94
0.88
0.96
0.90
0.96
0.97
0.88
0.95
0.94
0.98
0.95

Supplementary Figure 5. Correlation of housekeeping pathways in the MRST mutant vs NF54. Gene sets obtained from the Malaria Parasite Metabolic Pathways database corresponding to the following housekeeping pathways were analyzed for spearman correlation between the NF54 and MRST mutant timepoints: protein biosynthesis, DNA replication, transcriptional machinery, ribosome structure, fatty acid synthesis, redox metabolism, and genes coding for the proteasome. Spearman correlation values are shown between identical timepoints of NF54 and mutant, demonstrating transcriptional alignment at the metabolic pathway level. R was used to generate spearman correlation values and heatmaps, with spearman correlations available in Data Set S1 Tab 10.
