# Supplementary material for: A novel Modulator of Ring Stage Translation (MRST) gene alters artemisinin sensitivity in Plasmodium falciparum
Source: mSphere. 2023 May 23;8(4):e00152-23. doi: 10.1128/msphere.00152-23 (PMC10449512; doi:10.1128/msphere.00152-23)
Supplement: Fig S6 — GO 6 hrs. [file msphere.00152-23-s0006.pdf]

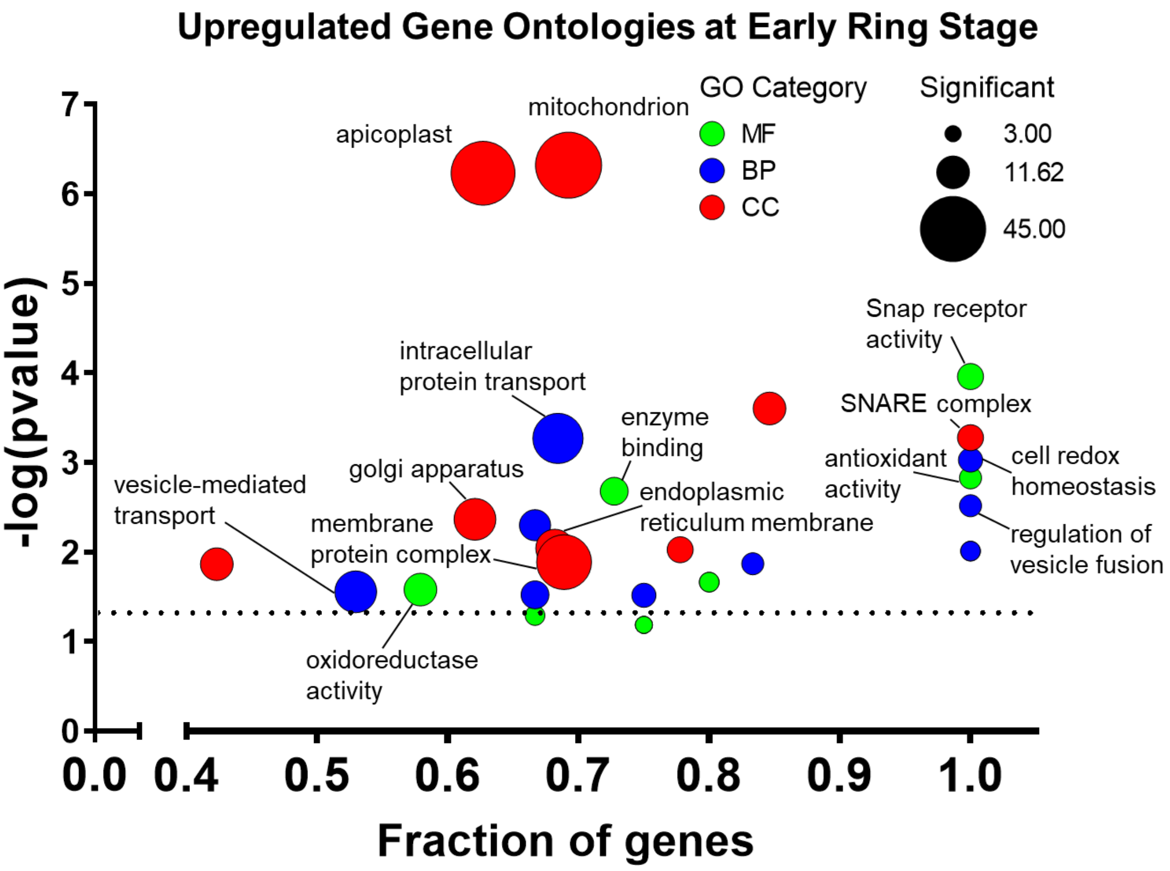
Upregulated Gene Ontologies at Early Ring Stage
GO Category
MF
BP
CC
Significant
3.00
11.62
45.00
apicoplast
mitochondrion
Snap receptor activity
intracellular protein transport
enzyme binding
SNARE complex
antioxidant activity
cell redox homeostasis
golgi apparatus
vesicle-mediated transport
endoplasmic reticulum membrane
membrane protein complex
regulation of vesicle fusion
oxidoreductase activity
-log(pvalue)
Fraction of genes
0
1
2
3
4
5
6
7
0.0
0.4
0.5
0.6
0.7
0.8
0.9
1.0

Supplementary Figure 6. Upregulated gene ontology enrichment terms of 6hpi expression in the MRST mutant. Gene ontology enrichment using the pfGO R package was performed on 6hpi MRST mutant gene dysregulation. Upregulated genes ontologies corresponding to molecular function (green), biological processes (blue), and cellular compartment (red) are shown, with the size of each bubble corresponding to the number of significant genes associated with each term in the mutant. GO term bubble plotted in GraphPad studio, with y-axis corresponding to -log(*p-value*) of term, and x-axis corresponding to the fraction of annotated genes present in significant DEGs. GO terms, genes lists associated with each term, along with *p-values* available in Data Set S1 Tab 12 and Tab 13.
