# Supplementary material for: A novel Modulator of Ring Stage Translation (MRST) gene alters artemisinin sensitivity in Plasmodium falciparum
Source: mSphere. 2023 May 23;8(4):e00152-23. doi: 10.1128/msphere.00152-23 (PMC10449512; doi:10.1128/msphere.00152-23)
Supplement: Fig S7 — tRNA ligase expression. [file msphere.00152-23-s0007.pdf]

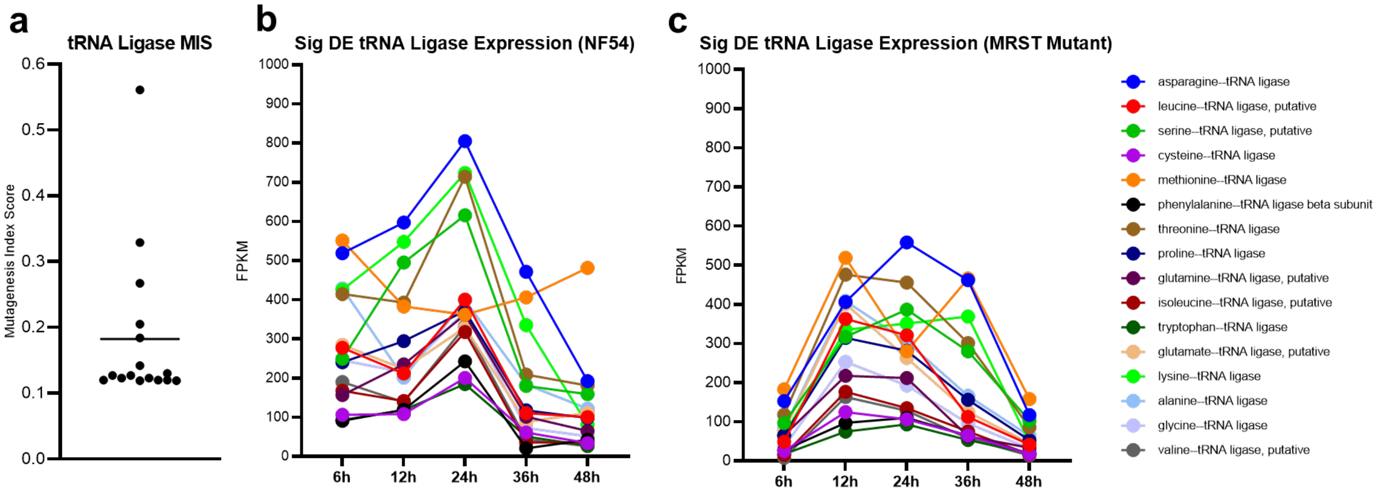

a
tRNA Ligase MIS
Mutagenesis Index Score
0.6
0.5
0.4
0.3
0.2
0.1
0.0
b
Sig DE tRNA Ligase Expression (NF54)
FPKM
1000
900
800
700
600
500
400
300
200
100
0
6h
12h
24h
36h
48h
c
Sig DE tRNA Ligase Expression (MRST Mutant)
FPKM
1000
900
800
700
600
500
400
300
200
100
0
6h
12h
24h
36h
48h
asparagine--tRNA ligase
leucine--tRNA ligase, putative
serine--tRNA ligase, putative
cysteine--tRNA ligase
methionine--tRNA ligase
phenylalanine--tRNA ligase beta subunit
threonine--tRNA ligase
proline--tRNA ligase
glutamine--tRNA ligase, putative
isoleucine--tRNA ligase, putative
tryptophan--tRNA ligase
glutamate--tRNA ligase, putative
lysine--tRNA ligase
alanine--tRNA ligase
glycine--tRNA ligase
valine--tRNA ligase, putative

Supplementary Figure 7. tRNA ligase expression patterns in NF54 and MRST mutant. Significant dysregulation of 16 tRNA ligase genes were identified at 6hpi in the mutant (*p-value* < 0.05) (Table 2). The mutagenesis index scores of these tRNA ligases are shown in (**a**), demonstrating the essentiality of the majority of these tRNA ligases at 6hpi in the mutant. FPKM expression patterns across the five timepoints sampled in this study of the 16 significantly dysregulated tRNA ligases are shown in NF54 (**b**) and the MRST mutant (**c**), further demonstrating differential expression patterns of tRNA ligases at 6hpi in the mutant. FPKM expression values are available in Data Set S1 Tab 4.
